# Post-Exposure Therapeutic Efficacy of COX-2 Inhibition against *Burkholderia pseudomallei*

**DOI:** 10.1371/journal.pntd.0002212

**Published:** 2013-05-09

**Authors:** Saja Asakrah, Wildaliz Nieves, Zaid Mahdi, Mallory Agard, Arnold H. Zea, Chad J. Roy, Lisa A. Morici

**Affiliations:** 1 Tulane University School of Medicine, Department of Microbiology and Immunology, New Orleans, Louisiana, United States of America; 2 Louisiana State University Health Sciences Center, Section of Pulmonary and Critical Care Medicine, New Orleans, Louisiana, United States of America; 3 Tulane National Primate Research Center, Division of Microbiology, Covington, Louisiana, United States of America; University of California San Diego School of Medicine, United States of America

## Abstract

*Burkholderia pseudomallei* is a Gram-negative, facultative intracellular bacillus and the etiologic agent of melioidosis, a severe disease in Southeast Asia and Northern Australia. Like other multidrug-resistant pathogens, the inherent antibiotic resistance of *B. pseudomallei* impedes treatment and highlights the need for alternative therapeutic strategies that can circumvent antimicrobial resistance mechanisms. In this work, we demonstrate that host prostaglandin E2 (PGE_2_) production plays a regulatory role in the pathogenesis of *B. pseudomallei*. PGE_2_ promotes *B. pseudomallei* intracellular survival within macrophages and bacterial virulence in a mouse model of pneumonic melioidosis. PGE_2_-mediated immunosuppression of macrophage bactericidal effector functions is associated with increased arginase 2 (Arg2) expression and decreased nitric oxide (NO) production. Treatment with a commercially-available COX-2 inhibitor suppresses the growth of *B. pseudomallei* in macrophages and affords significant protection against rapidly lethal pneumonic melioidosis when administered post-exposure to *B. pseudomallei*-infected mice. COX-2 inhibition may represent a novel immunotherapeutic strategy to control infection with *B. pseudomallei* and other intracellular pathogens.

## Author Summary

*Burkholderia pseudomallei* is the etiologic agent of melioidosis, a severe disease endemic in Southeast Asia and Northern Australia. *B. pseudomallei* is also classified as a Tier 1 select agent due to the threat of malicious use of the organism. Treatment of melioidosis is complicated by the inherent multidrug resistance of *B. pseudomallei*, leading to high case fatality rates or disease relapse. New therapeutic strategies are urgently needed to improve patient survival and to protect against a deliberate release of *B. pseudomallei*. Immunotherapeutics that can enhance the host immune response and delay disease progression represent a significant area of research interest. A number of immunomodulatory agents delivered locally to the lung prior to *B. pseudomallei* infection have afforded significant protection against pulmonary disease in animal models of melioidosis; however, their protective capacity significantly wanes upon post-exposure administration. In this work, we identify the PGE_2_ pathway as an immunotherapeutic target in pulmonary melioidosis and show that post-exposure COX-2 inhibition provides significant protection against lethal *B. pseudomallei* lung infection in mice. Further research examining FDA-approved COX-2 inhibitors as post-exposure prophylaxis for *B*. *pseudomallei* is warranted, as this may represent a safe, affordable, and efficacious immunotherapeutic strategy.

## Introduction

Development of new therapeutics effective against intracellular bacterial pathogens remains a high priority. In addition to the global impact of intracellular bacterial infections on public health, the alarming increase in multidrug resistant strains and the potential threat of biological attack with select agents, such as *Burkholderia pseudomallei*, highlight the urgent need for safe and effective therapies against this collective group of pathogens. *B*. *pseudomallei* is a Gram-negative, facultative intracellular bacillus and the causative agent of melioidosis, a disease associated with high morbidity and mortality in Southeast Asia and Northern Australia. Although melioidosis is not endemic in the United States, *B*. *pseudomallei* is classified as a Tier 1 select agent due to its ease of respiratory transmission, high mortality rate, multidrug resistance, and the absence of a protective vaccine [1]. Furthermore, malicious use of *B*. *pseudomallei* and *B. mallei* during World Wars I and II provides historical precedence for use of these agents as bioweapons and validates the need for post-exposure therapeutics that can be quickly administered to military personnel and civilians [2].

The inherent antibiotic resistance of *B*. *pseudomallei* limits chemotherapeutic options for melioidosis and the particular choice of antibiotic regimen has not been shown to impact mortality within the first 48 hours of hospitalization [3]. Current treatment requires intravenous administration of ceftazidime or meropenem, with or without trimethoprim-sulphamethoxazole (TMP-SMX), for two weeks of intensive phase therapy. The intensive phase of treatment may be extended up to eight weeks for deep-seated infections. Upon completion of this intensive phase, an eradication phase utilizing oral TMP-SMX or doxycycline for outpatient use is recommended for 8–12 weeks in order to prevent relapse. Despite this aggressive therapy, case fatality rates for severe melioidosis approach 40% in Thailand and 15% in Australia [4]. Therefore, it is necessary to develop new modalities of treatment that can replace or complement existing antibiotics to improve patient survival.

An appealing alternative as a first line therapeutic strategy is to enhance the host innate immune response during the early course of bacterial infection. In human trials, complementary use of granulocyte colony-stimulating factor improved the duration of survival for melioidosis patients with severe sepsis but did not decrease mortality rates [5]. In pre-clinical studies, treatment of BALB/c mice with cationic liposomal DNA complexes (CLDC) 24 h prior to intranasal *B*. *pseudomallei* challenge enhanced natural killer (NK) cell recruitment and afforded complete protection from a lethal infectious dose [6]. Similarly, treatment of BALB/c mice with the TLR9 agonist, CPG ODN, 48 h prior to *B*. *pseudomallei* infection led to significantly lower tissue bacterial burdens and improved overall survival [7], [8]. Combining vaccination with CpG treatment that was given up to 18 h post-infection provided significantly greater protection against *B*. *pseudomallei* than either treatment alone, indicating that immune modulation with CpG can also enhance the efficacy of other countermeasures [9]. In contrast, post-exposure prophylaxis with CpG alone was not effective against *B*. *pseudomallei*, as initial control of bacterial growth appears dependent upon prior recruitment of inflammatory cells to the lung [8]. Since bacterial infection cannot be predicted, it is imperative to identify immunotherapeutics that can mediate protection when administered post-exposure.

In the present study, we identify the prostaglandin E2 (PGE_2_) pathway as a novel therapeutic target during pneumonic melioidosis. PGE_2_ is a potent lipid mediator derived from cyclooxygenase (COX) metabolism of the cell membrane fatty acid, arachidonic acid [10]. PGE_2_ is produced in response to inflammation via the COX-2 enzyme and is a key mediator of immunopathology in chronic disease, autoimmunity, and cancer [10]. While PGE_2_-mediated immunoregulation is essential for maintaining homeostasis, its suppressive effects on innate and adaptive immunity may be counter-productive during infection. In this work, we demonstrate that *B*. *pseudomallei* rapidly induces macrophage COX-2 expression and PGE_2_ production which establishes a permissive environment for *B*. *pseudomallei* intracellular persistence. Pulmonary infection with *B*. *pseudomallei* leads to increased concentrations of lung PGE_2_, and lung PGE_2_ levels significantly correlate with disease progression in mice. Post-exposure administration of a COX-2 inhibitor provides significant protection against lethal pulmonary challenge with *B*. *pseudomallei*. This is the first demonstration of a non-antibiotic post-exposure therapeutic that provides significant protection on its own against lethal pulmonary infection with *B*. *pseudomallei*. Therapeutic strategies targeting the PGE_2_ pathway may delay disease progression in pneumonic melioidosis and afford a window of opportunity for antibiotic intervention and/or development of adaptive immunity. Furthermore, COX-2 inhibition may represent a novel and universal immunotherapeutic strategy against other intracellular pathogens.

## Materials and Methods

### Mice and Bacterial Challenges

Ethics Statement: Animal experiments were performed in strict accordance with the recommendations in the Guide for the Care and Use of Laboratory Animals of the National Institutes of Health. The protocol was approved by the Tulane University Institutional Animal Care and Use Committee (protocol number 4042). Six to eight week old, female BALB/c mice (Charles River) were maintained under pathogen-free conditions and fed sterile food and water *ad libitum*. Infections utilizing *Bps* were performed under Animal Biosafety Level 3 containment.

*Burkholderia pseudomallei* strain 1026b (BEI Resources) was used in this study. For infectious challenge, mice were anesthetized with Ketamine/xylazine (88 mg/kg) (Fort Dodge Animal Health). The bacterial inoculum contained 3×10^3^ cfu (_∼_4 LD_50_) suspended in 40 µl sterile saline and 20 µL was delivered to each nostril via pipet. Bacterial cfu were confirmed by plating the inoculum on LB agar. Euthanasia endpoints used in this study included loss of >20% body weight, hunched posture and decreased movement or response to stimuli, or paralysis. In a subset of experiments, mice were treated with the selective COX-2 inhibitor, (*N*-[2-(cyclohexyloxy)-4-nitrophenyl]-methanesulfonamide) (NS398) (Cayman Chemicals), 3 h post-exposure. Mice received 50 µl of NS398 (15 mg/kg) dissolved in DMSO or vehicle control (DMSO) by intraperitoneal injection. Treatments were repeated for two consecutive days. After euthanasia, tissues were removed, weighed and homogenized in 1 ml 0.9% sterile saline. Serial dilutions of tissue homogenates were plated on LB agar and bacterial cfu were counted after 2–4 days of incubation at 37°C.

### Cell Culture and *In Vitro* Experiments

J774A.1 murine macrophage-like cells were obtained from ATCC. Cells were propagated in media containing DMEM (Invitrogen) with 10% FBS (Atlanta Biologicals), 1% Pen/Strep (Invitrogen) and 1% sodium bicarbonate (Invitrogen). Bone marrow-derived macrophages (BMDM) were extracted from 8–10 week old BALB/c mice as previously described [11]. BMDM were propagated in RPMI (ATCC), containing 15% L929 fibroblast-conditioned media, 2 g/L D-glucose (Invitrogen), 10% FBS, 5% horse serum (Invitrogen), 1% Pen/Strep and 2 mM L-glutamine (Invitrogen). Prior to each experiment, the cytotoxic dose of bacteria and chemical treatments were pre-determined using a colorimetric assay for LDH release (Clontech). Intracellular survival assays were performed as previously described [12]. In some experiments, cells were treated with 100 µM of NS398, 100 µM nor- N^ω^-hydroxy-L-arginine (nor-NOHA; Cayman Chemical), or 1 µM PGE_2_ (Sigma). PGE_2_ was measured in cell culture supernatants and lung homogenates by competitive ELISA (Pierce). Nitric oxide was measured as its stable end product nitrite by Griess assay (Invitrogen).

### TLR Pathway PCR Array

Fold-change in mRNA expression of 84 genes central to TLR-mediated signal transduction and innate immunity were measured by PCR array following the manufacturer's protocol and data analysis software (SABiosciences).

### Real Time-PCR

RT-PCR was conducted using an iCycler (BioRad) with iScript cDNA Synthesis Kit (BioRad). 1 µg of RNA was converted to cDNA following the manufacturer's protocol. 1 µl of cDNA was added to 12.5 µL of iQ SYBR Green Super Mix containing 350 nM of each forward and reverse primer. Primer sequences were as follows: GAPDH: forward, 5′-ACAGCCGCATCTTCTTGTGCAGTG-3′; reverse, 5′-GGCCTTGACTGTGCCGTTGAATTT-3′; Arg1: forward, 5′-GGGCTGGACCCAGCATTCACCCCG-3′; reverse, 5′TCACTTAGGTGGTTTAAGGTAGTC-3′; Arg2: forward, 5′-GACCCTAAACTGGCTCCAGCCACA-3′; reverse, 5′-CTAAATTCTCACACATTCTTCATT-3′; iNOS: forward, 5′-ATGACCAGTATAAGGCAAGC-3′; reverse, 5′-GCTCTGGATGAGCCTATATTG-3′; COX-2: forward, 5′-GGAGAGAAGGAAATGGCTGCA-3′; reverse, 5′-ATCTAGTCTGGAGTGGGAGG-3′. Nuclease-free water was added to bring the total reaction volume to 25 µL. PCR was performed using the following conditions: reverse transcriptase inactivation (95°C, 3 min) followed by 40 PCR cycles (95°C, 15 seconds and 60°C, 30 seconds) followed by melt curve analysis. Fold change (up- or down- regulation) relative to base line expression in uninfected cells was calculated using the ΔΔC_t_ method using C_t_ values for arginase 1, arginase 2, iNOS, COX-2, and GAPDH.

### Detection of COX-2 and Arginase

For Western blot, equal amounts of protein (50 µg) from cell lysates or lung homogenates were resolved by SDS-PAGE and transferred to nitrocellulose using an iBLOT (Invitrogen). Detection of COX-2 enzyme was performed using a 1∶1000 dilution of rabbit polyclonal anti-COX-2 (Cell Signaling Technology), followed by peroxidase-conjugated, donkey anti-rabbit IgG (1∶5000). Arg2 was detected using a 1∶200 dilution of rabbit polyclonal anti-mouse Arg2 (sc-20151, Santa Cruz) and Arg1 was detected using a 1∶500 dilution of Arg1 antibody 18351 (Santa Cruz). Mouse Arg2-transfected 293T cell lysate (Santa Cruz) was used a positive control for Arg2 and 5 µg of mouse liver extract was used a positive control for Arg1. β-actin, a protein loading control, was detected using 1∶1000 dilution of polyclonal rabbit anti-mouse β-actin (Cell Signaling). Detection of bound antibodies was visualized by a chromogenic reaction using Opti-4CN Substrate (BioRad).

### Statistical Analysis

Statistical analyses were performed using Prism 5.0 software (Graph Pad). Kaplan–Meier survival curves were compared by log-rank analysis. All other data were analyzed using a one-way or two-way ANOVA followed by the Bonferroni post-test to determine statistical differences between groups. p<0.05 was considered statistically significant. All data are representative of at least two independent experiments.

## Results

### *B. pseudomallei* Rapidly Induces PGE_2_ Production by Macrophages

*B*. *pseudomallei* is remarkable in its ability to establish chronic infection that can reactivate decades after the initial infection and yet virtually nothing is known regarding the mechanisms by which *B*. *pseudomallei* evades immune clearance [1]. In order to identify host cell signaling pathways that might contribute to *B*. *pseudomallei* intracellular persistence, we performed a Toll-like receptor (TLR) PCR array on J744A.1 macrophages infected with *B. thailandensis*. *B. thailandensis* is a commonly used biosafety level 2 surrogate organism for the study of *B*. *pseudomallei* and, with the exception of capsular polysaccharide, possesses all of the known *B*. *pseudomallei* virulence determinants such as Type 3 and Type 6 secretion systems [13]–[17]. Although *B. thailandensis* is 1,000- to 100,000-fold less virulent than *B*. *pseudomallei* in animal models, the organisms behave very similarly *in vitro*. *B. thailandensis* and *B*. *pseudomallei* induce pyroptosis in macrophages as early as 8 h post infection at a multiplicity of infection (MOI) 10 or greater [18]. In pilot experiments, we determined that J774A.1 macrophages infected with *B. thailandensis* at MOI 10 or 1 displayed 80% and 28% cytotoxicity, respectively at 8 h post-infection (not shown). Therefore, experiments utilizing J774A.1 macrophages or primary bone marrow-derived macrophages (BMDM) were limited to an eight hour experimental time course using *B. thailandensis* or *B*. *pseudomallei* at MOI 1 or lower (0.1).

Consistent with previous reports [19]–[22], *B. thailandensis* upregulated expression of TLR1 and TLR2 by two h post-infection, and increases in TLR1, TLR2, TLR3, TLR4, and TLR5 mRNA expression were observed by eight h post-infection (Supporting information, Table S1). No change in mRNA expression was observed for TLR6, 7, 8, or 9. One of the most striking changes in expression occurred in COX-2, the enzyme responsible for the production of PGE_2_. A rapid increase (430-fold) in COX-2 mRNA expression occurred by two h post-infection and further increased by >16,000-fold at eight h (Supplementary Table S1).

**Figure 1:**
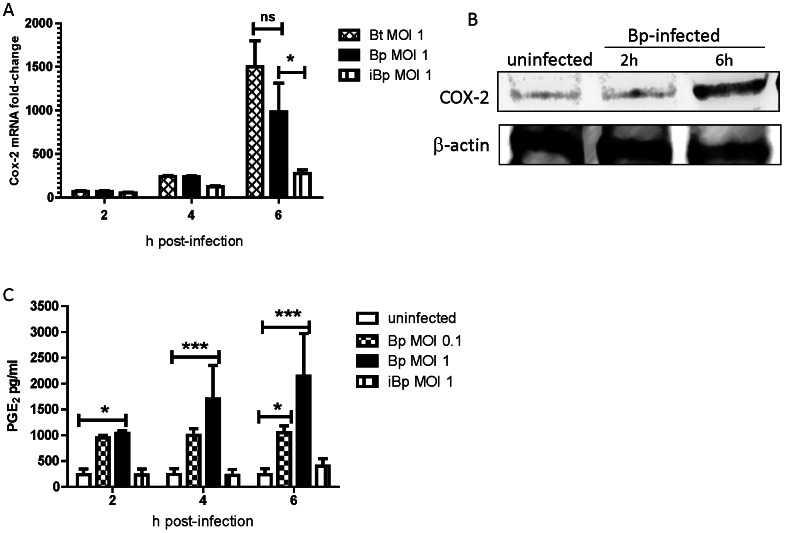
*B.* pseudomallei rapidly induces COX-2 and PGE_2_ production by macrophages. **A**) Bone-marrow derived macrophages (BMDM) were treated with viable *B. thailandensis* (Bt), *B. pseudomallei* (Bp) or heat-inactivated Bp (iBp) at MOI 1 and COX-2 mRNA expression was measured by RT-PCR. **B**) COX-2 enzyme was detected by Western blot in BMDM infected with Bp at MOI 1. **C**) BMDM were treated with Bp at MOI 0.1 or 1 and iBp at MOI 1 and PGE_2_ was measured in culture supernatants by ELISA. The data represent biological triplicates per time point. Error bars represent the standard deviation (SD). Statistical significance was determined using a two way ANOVA with Bonferroni post-test. *p<0.05, *** p<0.001. Data is representative of two independent experiments.

To confirm the TLR array results obtained for *B. thailandensis*-infected J774A.1 macrophages, BMDM were infected with *B. thailandensis* and *B*. *pseudomallei* (MOI 1) and COX-2 mRNA expression was measured by RT-PCR. *B. thailandensis* and *B*. *pseudomallei* both up-regulated COX-2 mRNA expression in BMDM to a similar extent (Fig. 1A), although the levels of mRNA expression were lower than that observed for *B. thailandensis*-infected J774A.1 cells and may reflect differences between immortalized and primary cell lines (Fig. 1A, Supplementary Table S1). COX-2 enzyme and its end product, PGE_2_, were also produced by macrophages in response to *B*. *pseudomallei* in a time- and dose-dependent manner (Fig. 1B–C). Since lipopolysaccharide (LPS) of Gram-negative bacteria is known to induce COX-2 and PGE_2_ production, we evaluated whether the PGE_2_ response of infected macrophages was simply a passive signaling event mediated by TLR4 recognition of LPS. Notably, heat inactivation of *B*. *pseudomallei* significantly abolished COX-2 and PGE_2_ expression (Fig. 1A–C) indicating that viable bacteria and/or bacterial proteins are required for early PGE_2_ production by macrophages.

### PGE_2_ Enhances *B. pseudomallei* Intracellular Survival

**Figure 2:**
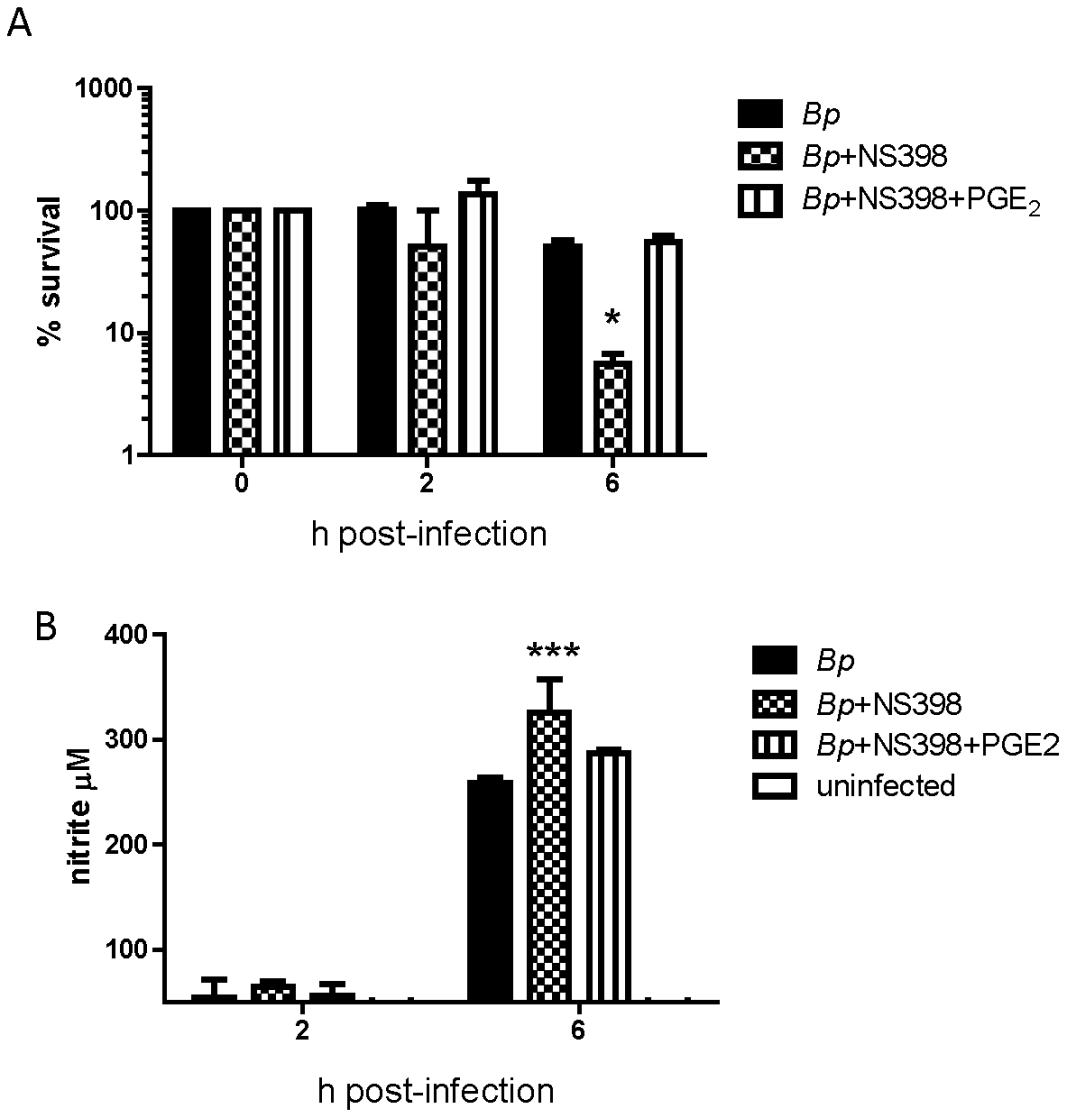
PGE_2_ promotes *B.* pseudomallei intracellular survival. BMDM were incubated in the presence or absence of NS398 (100 µM) +/− PGE_2_ (1 µM) for 30 minutes then infected with *Bps* at MOI 1 **A**) Percent intracellular survival of *Bps* and **B**) corresponding nitrite levels in BMDM supernatants. The data represent biological triplicates per time point. Error bars represent the SEM. Statistical significance was determined using a two way ANOVA with Bonferroni post-test. *p<0.05, *** p<0.001. Data is representative of two independent experiments.

Because PGE_2_ has been shown to suppress macrophage bactericidal mechanisms [23], we assessed the impact of COX-2 activation and PGE_2_ production on *B*. *pseudomallei* intracellular survival using the selective COX-2 inhibitor, NS398. Preliminary dose-response experiments were conducted using 10 to 200 µM NS398 (not shown). BMDM treated with ≥100 µM NS398 demonstrated enhanced intracellular killing of *B*. *pseudomallei* compared to non-treated cells by six h post-infection (Fig. 2A). To verify the specificity of NS398 and that endogenous PGE_2_ is responsible for the suppression of bacterial killing, exogenous PGE_2_ was added to NS398-treated cells. Addition of PGE_2_ to the cell cultures restored *B*. *pseudomallei* intracellular survival (Fig. 2A) confirming that PGE_2_ promotes a favorable environment for *B*. *pseudomallei*.

Previous work has shown that macrophage bactericidal activity against *B*. *pseudomallei* is mediated to a large extent by reactive nitrogen species and to a lesser extent by reactive oxygen species (ROS) [24], [25]. PGE_2_ has been shown to suppress nitric oxide (NO) synthesis in Kupffer cells, hepatocytes, murine peritoneal macrophages, and RAW 264.7 murine macrophages [26]. Therefore, we evaluated the downstream effect of PGE_2_ on the macrophage NO response to *B*. *pseudomallei* infection. Treatment of BMDM with the COX-2 inhibitor NS398 led to a significant increase in nitrite, the stable end product of NO (Fig. 2B). This effect was not drug-specific because similar results were obtained using the COX inhibitor, indomethacin (not shown). Conversely, the addition of exogenous PGE_2_ to NS398-treated macrophages significantly reduced nitrite levels in *B*. *pseudomallei*-infected cells (Fig. 2B). This suggests that PGE_2_-mediated suppression of NO production may partially contribute to *B*. *pseudomallei* intracellular survival.

### Arginase 2 Enhances *Bps* Intracellular Survival

**Figure 3:**
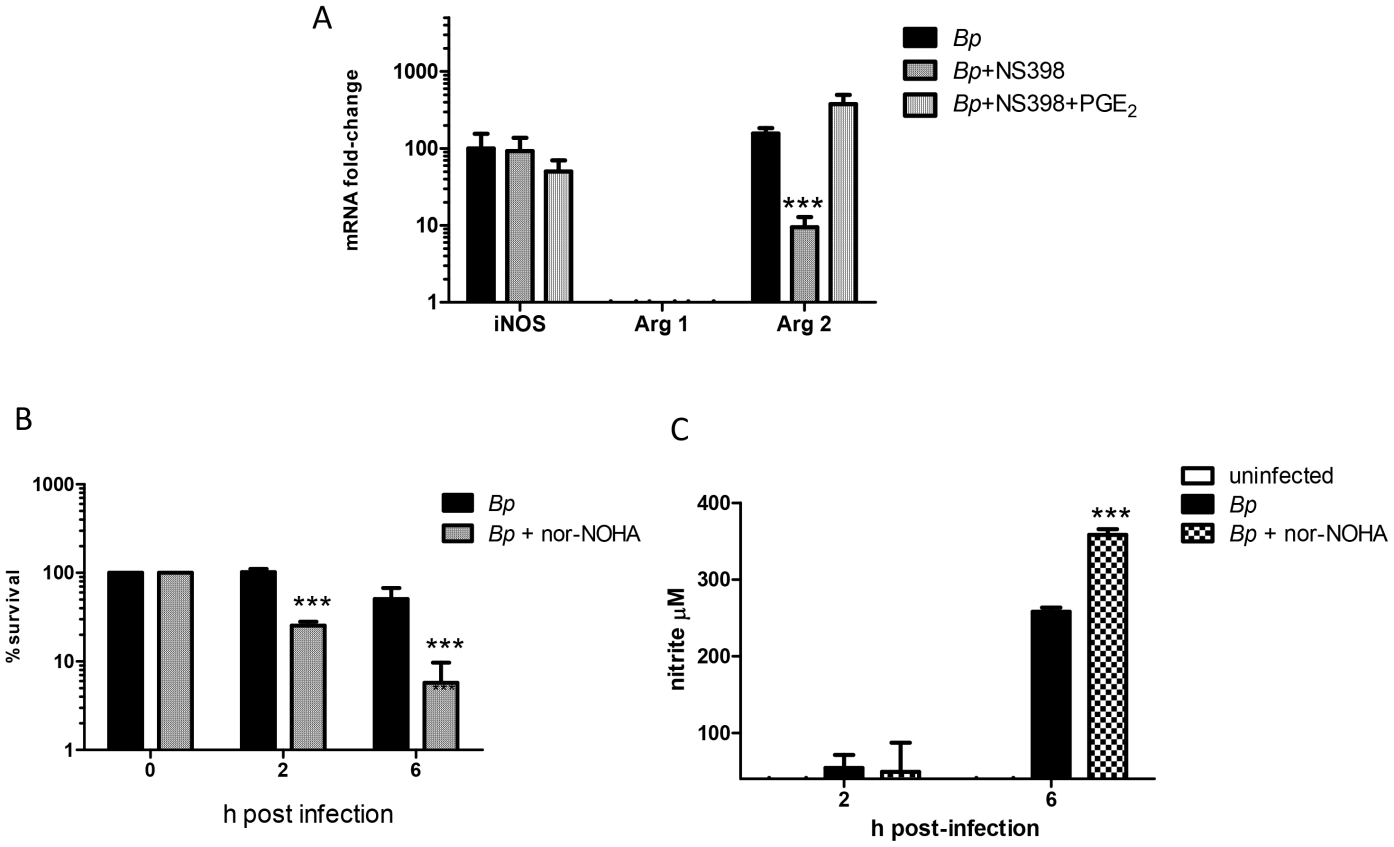
Arginase 2 enhances *B.* pseudomallei survival in macrophages. BMDM were incubated in the presence or absence of NS398 (100 µM) +/− PGE_2_ (1 µM) for 30 minutes, then infected with *Bps* at MOI 1 for 4 h **A**) Fold-change in mRNA expression for iNOS, arginase 1 (Arg1) and Arg2 in response to *Bps* was measured by RT-PCR. Error bars represent the SEM. **B**) Intracellular survival of *Bps* in BMDM pre-treated with 100 µM nor-NOHA for 30 minutes and **C**) corresponding nitrite production by *Bps*-infected cells in the presence or absence of nor-NOHA. The data represent biological triplicates per time point. Error bars represent the SEM. Statistical significance was determined using a two way ANOVA with Bonferroni post-test. *** p<0.001. Data is representative of two independent experiments.

We next examined the effect of endogenous PGE_2_ production on the expression of iNOS, which is required for the synthesis of NO. We did not observe any significant change in iNOS mRNA expression in NS398- or PGE_2_-treated cells compared to controls infected with *B*. *pseudomallei* (Fig. 3A). This suggested that PGE_2_ did not directly regulate iNOS in *B*. *pseudomallei*-infected cells and that other mechanisms were responsible for the reduced levels of NO.

Since the enzymes arginase 1 (Arg1) and 2 (Arg2) compete with iNOS for the substrate, L-arginine, we postulated that PGE_2_ induction of arginase could alter the level of NO production during *B*. *pseudomallei* infection. PGE_2_ induction of macrophage arginase promotes tumor cell growth by suppressing NO-mediated tumor cytotoxicity [27], [28]. Arg1 expression was not detected after four h of *B*. *pseudomallei*-infection, but the expression of Arg2 was significantly increased (155-fold) in *B*. *pseudomallei-*infected BMDM (Fig. 3A). NS398-treated macrophages demonstrated a significant reduction in Arg2 expression while treatment with exogenous PGE_2_ increased Arg2 expression by 376-fold (Fig. 3A). These data suggest that endogenous PGE_2_ may interfere with NO production by enhancing Arg2 expression.

Modulation of the arginase pathway contributes to the intracellular survival of multiple pathogens, including *Salmonella* and *Mycobacterium* spp. [29]. To determine whether Arg2 directly interferes with NO production and enhances *B*. *pseudomallei* intracellular survival, we treated macrophages with the arginase inhibitor, nor-NOHA. A significant decrease in *B*. *pseudomallei* intracellular survival was observed in nor-NOHA-treated BMDM (Fig. 3B) and this corresponded to a significant increase in nitrite levels (Fig. 3C). Collectively, these results indicate that Arg2 expression promotes *B*. *pseudomallei* intracellular survival, in part, through suppression of macrophage NO synthesis.

### PGE_2_ is Produced during *Bps* Pulmonary Infection

**Figure 4:**
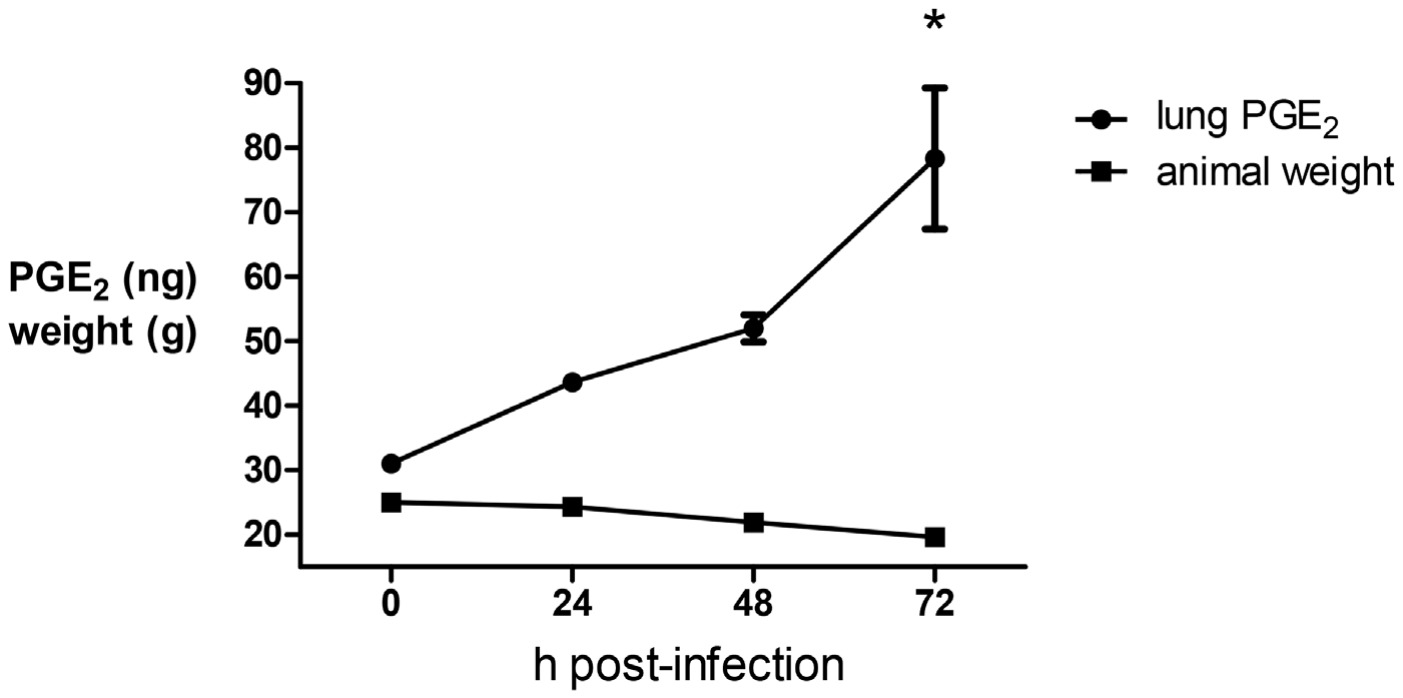
Lung PGE_2_ increases in a time-dependent manner after pulmonary challenge with *B.* pseudomallei. BALB/c mice were infected intranasally with 3×10^3^ cfu of *Bps* and serially sacrificed between 0 (pre-challenge) and 72 h post-infection (n = 3 per timepoint). Animal weight was recorded daily and PGE_2_ was measured in total lung homogenates by ELISA. Error bars represent the SEM. *p<0.05 compared to 0 h timepoint as determined by one way ANOVA. Data is representative of two independent experiments.

Inhalational infection with *B*. *pseudomallei* is a natural route of exposure and represents the most likely route of infection in a deliberate biological attack [2]. In order to evaluate the role of PGE_2_ during pneumonic melioidosis, genetically-susceptible BALB/c mice were challenged by the intranasal route with a lethal dose of *B*. *pseudomallei* (3×10^3^ cfu) [30]. Pulmonary infection with *B*. *pseudomallei* progressed rapidly in mice leading to greater than 20% weight loss by 72 h post-infection (Fig. 4). A significant increase in lung PGE_2_ was observed by 72 h post-infection and significantly correlated with disease progression (p = 0.029 by Pearson statistical analysis) (Fig. 4). These results indicate that PGE_2_ may play an important role in pneumonic melioidosis during the early stages of infection.

### Protective Efficacy of COX-2 Inhibition against Pneumonic Melioidosis

**Figure 5:**
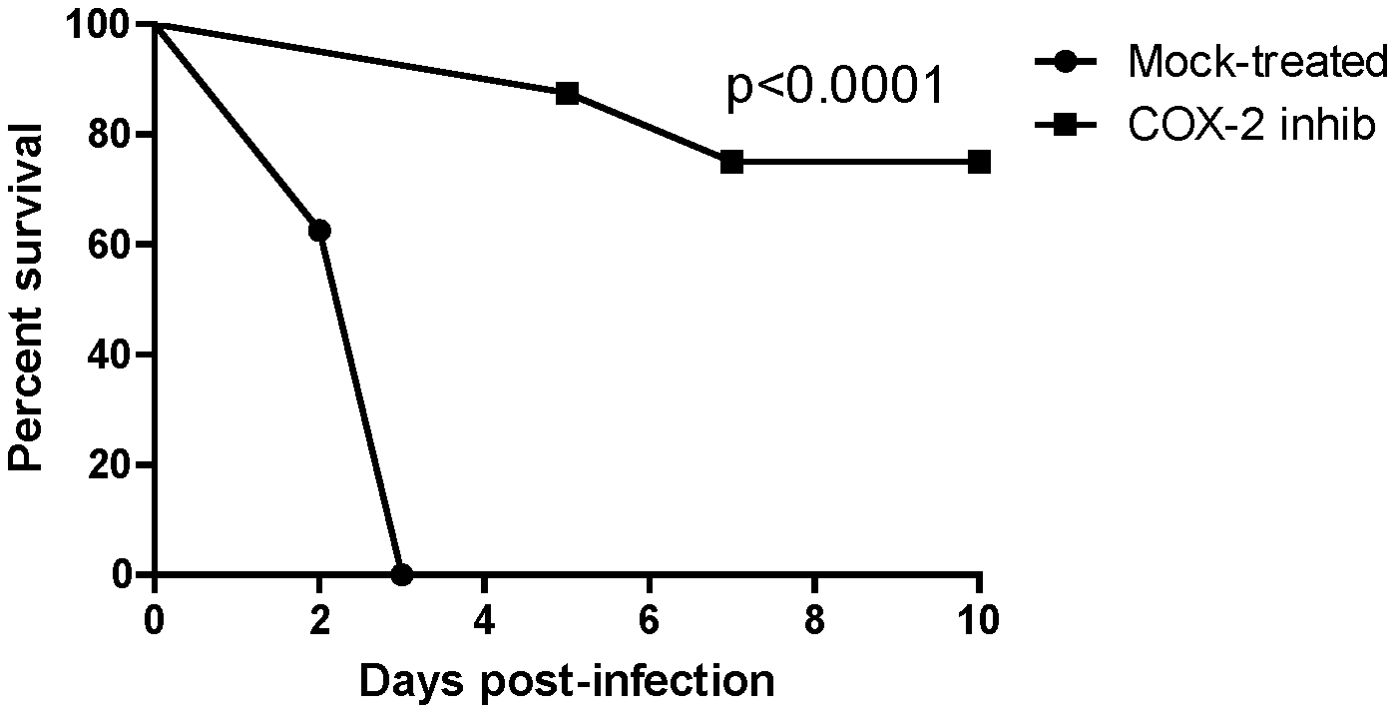
COX-2 inhibition provides significant protection against lethal pulmonary melioidosis. BALB/c mice (n = 8 per group) were infected with 3×10^3^ cfu (4 LD_50_) of *Bps* 1026b intranasally. Three h post-exposure, mice were administered 15 mg/kg of COX-2 inhibitor (NS398) or DMSO (Mock treatment) intraperitoneally, then again daily for two consecutive days. Survival was monitored for 10 days. Statistical significance was determined using Kaplan Meier analysis. p<0.0001. Data is representative of two independent bacterial challenge experiments.

Because PGE*_2_* inhibition enhanced bacterial clearance *in vitro* and because PGE_2_ is elevated in *B*. *pseudomallei*-infected lungs, we evaluated the efficacy of COX-2 inhibition as a post-exposure therapeutic strategy against lethal *B*. *pseudomallei* pulmonary challenge. Mice were given NS398 or mock control by i.p. administration three h after *B*. *pseudomallei* intranasal infection, and treatments were repeated for two consecutive days. Initiation of therapy within three h is clinically relevant in the case of a known biological exposure to *B*. *pseudomallei*, such as a laboratory accident. A daily maximum dose of 15 mg/kg of NS398 was selected based upon previously documented pharmacological efficacy in mice (particularly in reducing lung PGE_2_) without any associated toxicity [31]. Mock-treated mice infected with *B*. *pseudomallei* rapidly displayed signs of pulmonary disease and all had to be euthanized within 72 h (Fig. 5). Lungs of mock-treated mice all contained greater than 10^6^ cfu of *B*. *pseudomallei* at the time of euthanasia. In contrast, none of the NS398-treated mice showed signs of illness until day 5 post-infection. On day 5, one mouse in the NS398-treated group displayed hind leg paralysis and was humanely euthanized. This was observed again in another animal on day 7. No bacteria were recovered from the lungs of either animal. Intranasal infection of mice with *B*. *pseudomallei* often manifests in colonization of the brain with subsequent neurologic complications [30], and we believe that this, and not pulmonary disease, likely accounted for the animals' morbidity. By day 10, all of the remaining NS398-treated mice appeared to have recovered from the infection. NS398-treated mice showed no evidence of weight loss throughout the study (not shown). No bacteria were recovered from the lungs of NS398-treated mice at the study endpoint with the exception of one animal that contained 10^4^ cfu. All of the mice were colonized with 20–100 cfu in the spleen and liver, indicating that bacterial dissemination from the lung had occurred. These results indicate that host PGE_2_ production promotes the pathogenesis of *B*. *pseudomallei* during pneumonic melioidosis and that inhibition of COX-2 enhances bacterial clearance from the lung and improves host survival. Consistent with these findings, COX-2 inhibition also significantly reduced tissue bacterial burdens and pulmonary inflammation in mice infected with *B. thailandensis* (Supporting information, Figures S1, S2).

### Effect of COX-2 Inhibition on Lung Arginase

**Figure 6:**
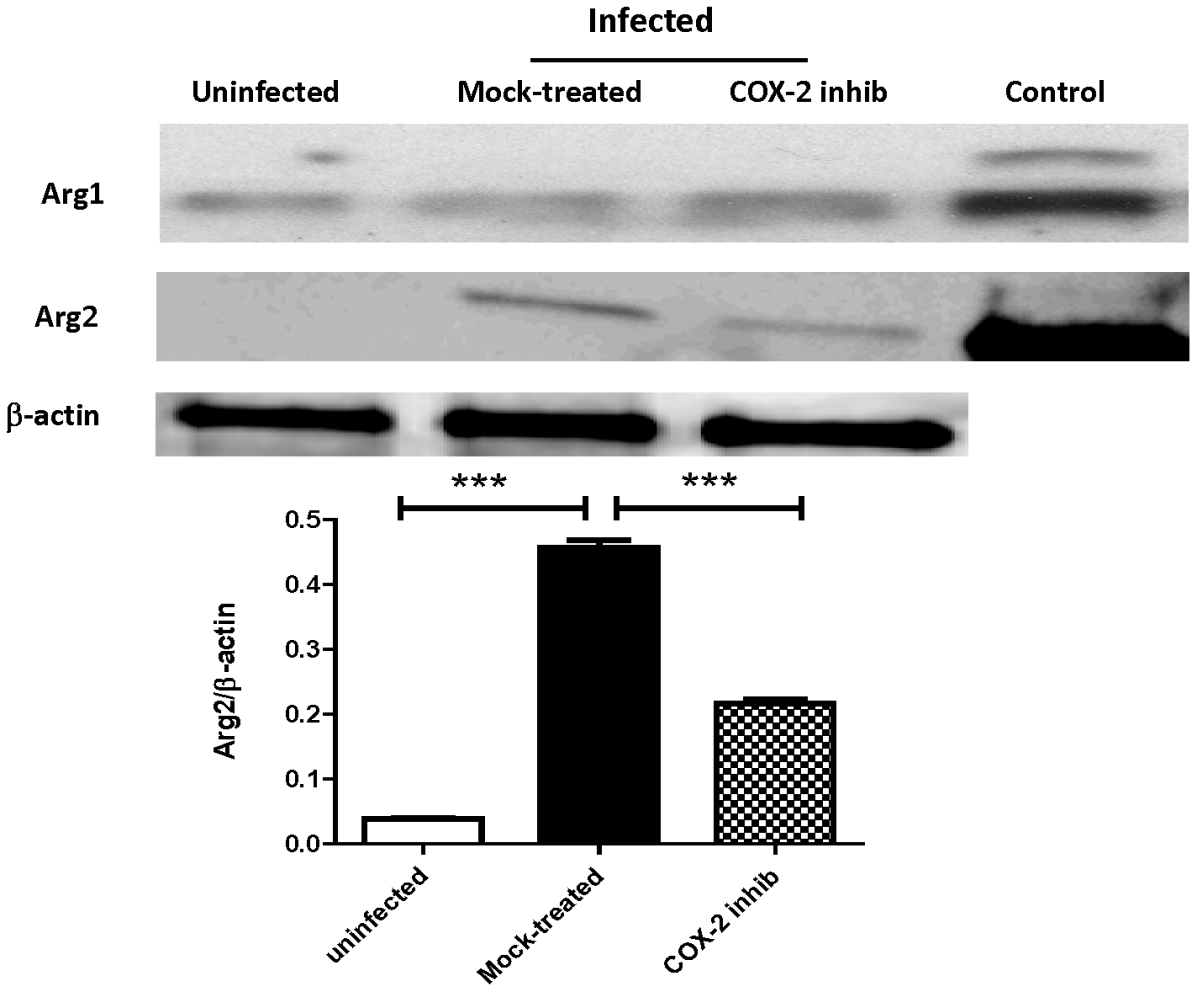
Arg2 is expressed in the lungs of *B.* pseudomallei-infected mice and decreases upon COX-2 inhibition. Arg1 and Arg2 expression was examined by Western blot in lung homogenates of uninfected and *Bps*-infected mice (n = 3 per group) treated with NS398 or mock control. Mouse liver extract and mouse Arg2-transfected 293T cell lysate were used as positive controls for Arg1 and Arg2 respectively. β-actin was used as a loading control and for normalization in densitometry analysis using the ImageJ program: http://rsb.info.nih.gov/ij/. Statistical significance was determined using one-way ANOVA with Bonferroni post test. *** p<0.001.

*B*. *pseudomallei* infection led to increased PGE_2_ and Arg2 expression in macrophages and both PGE_2_ and Arg2 enhanced *B*. *pseudomallei* intracellular survival. Furthermore, PGE_2_ positively regulated Arg2 expression in response to *B*. *pseudomallei in vitro*. We therefore evaluated Arg2 expression in the lungs of mice in response to bacterial infection and COX-2 inhibition. Similar to our *in vitro* observations, an increase in lung Arg2, but not Arg1, was observed in *B*. *pseudomallei*-infected animals compared to uninfected animals (Fig. 6). Upon COX-2 inhibition, a reduction in lung Arg2 was observed in *B*. *pseudomallei*-infected mice as evident by Western blot and densitometry analysis (Fig. 6). These results corroborate our observations in murine macrophages and advocate a supporting role for Arg2 in PGE_2_-mediated immunosuppression during *B*. *pseudomallei* infection.

## Discussion

Enhancement of non-specific innate immunity represents an attractive therapeutic strategy to combat infection with multidrug resistant bacterial pathogens. In this study, we demonstrate a critical role for PGE_2_ in the early pathogenesis of *B*. *pseudomallei* pulmonary infection and identify the PGE_2_ pathway as an immunotherapeutic target in melioidosis. PGE_2_ can negatively regulate innate immunity by suppression of leukocyte activation [32], macrophage microbicidal activity [23], and NK cell function [33]. Over-production of PGE_2_ has also been observed in a number of clinical conditions associated with an increased susceptibility to bacterial infection, including AIDS [34]. Therefore, the results presented here may be applicable to other intracellular bacteria, particularly those that infect the lung. PGE_2_ is a potent pro-inflammatory mediator in most tissues but plays an opposite role in the lung and gastric mucosa in order to limit inflammation and tissue injury upon mucosal insult [35], [36]. PGE_2_ concentrations in the lung are much higher than in plasma [37] so the bacterial survival advantage afforded by PGE_2_ in the present study may be exclusive to pulmonary infection and requires further study.

Macrophages play an important role in early host defense against *B*. *pseudomallei* as macrophage-depleted mice display an accelerated mortality during experimentally-induced melioidosis [38]. Our *in vitro* studies demonstrated that endogenous and exogenous PGE_2_ promoted *B*. *pseudomallei* intracellular survival, while inhibition of COX-2 eliminated endogenous PGE_2_ production and restricted bacterial growth in macrophages. The therapeutic efficacy of COX-2 inhibition at the cellular level may account for its significant protective efficacy against pulmonary melioidosis when administered three h after *B*. *pseudomallei* challenge. A number of studies have shown the clinical effectiveness of various immunostimulants delivered intranasally to mice prior to *B*. *pseudomallei* pulmonary challenge but none have demonstrated significant protective efficacy as a stand-alone post-exposure therapeutic [6], [8], [9], [39]. For example, CpG ODN was ineffective when given as early as one h after intranasal *B*. *pseudomallei* challenge due to a delay in recruitment of inflammatory monocytes and neutrophils to the lung [8]. Unlike these studies, the COX-2 inhibitor was administered intraperitoneally to mice after *B*. *pseudomallei* pulmonary delivery, indicating that its efficacy does not rely upon local administration and subsequent inflammatory cell recruitment. We have not yet assessed the post-exposure window of efficacy for COX-2 inhibition but we postulate that its direct action on infected macrophages, with less dependence on additional phagocyte recruitment, may allow a greater time frame for therapeutic intervention than we have already shown.

PGE_2_-mediated suppression of macrophage bactericidal ability has been observed in other bacterial pulmonary infections and is not restricted to the mouse model. For example, pre-treatment of rat alveolar macrophages with the COX-1/2 inhibitor, indomethacin, or antagonists of the PGE_2_ receptors, EP-2 and EP-4, augmented NADPH oxidase and ROS production and improved killing of *Klebsiella pneumoniae* [23]. It has been proposed that the timing and concentration of PGE_2_ determines the macrophage bactericidal response to stimuli such as IFN-γ and LPS [40]. Therefore, rapid production of high concentrations of PGE_2_ in response to *B*. *pseudomallei* may suppress macrophage control of bacterial growth early in infection. While our results demonstrated that PGE_2_ suppressed NO and enhanced Arg2, the mechanism(s) by which PGE_2_ exerts its suppressive effects on *B*. *pseudomallei*-infected macrophages may involve inhibition of additional reactive oxygen [23], [41] and nitrogen species [42] or pro-inflammatory cytokines [43] and warrants further study. Nonetheless, our studies add *B*. *pseudomallei* to the growing list of intracellular pathogens that utilize host arginase to facilitate their survival [29]. PGE_2_-mediated immunosuppression through arginase induction is well-established in cancer [44]. Our study highlights a similar mechanism operating during an intracellular bacterial infection. The clinical benefit of COX-2 inhibitors against lung carcinoma [44] and other cancers [10], [45] lends support to the potential use of this class of inhibitors against respiratory bacterial infection.

Interestingly, heat-inactivated *B*. *pseudomallei* did not induce appreciable levels of COX-2 expression or PGE_2_ production by macrophages indicating that the response is not stimulated by bacterial LPS. Although bacterial flagellin has been shown to induce COX-2 expression through TLR5 recognition and p38 MAPK signaling [46], *P. aeruginosa* strains lacking flagellin induce COX-2 expression comparable to wild type strains [41]. In addition, the majority of our *in vitro* studies were performed using murine BMDM which have been shown to be unresponsive to flagellin due to an absence of TLR5 [47] [48]. Collectively, these findings suggest that the macrophage PGE_2_ response is predominantly regulated by active bacterial processes as opposed to a passive signaling event mediated by bacterial PAMPs. In support of this view, COX-2 expression in the lungs of mice infected with *Pseudomonas aeruginosa* was dependent upon viable bacteria and the presence of the type three secretion system effector, ExoU, a member of the phospholipase A family [41]. Although the *B*. *pseudomallei* effector(s) responsible for the induction of PGE_2_ remain to be identified, it is plausible that secreted bacterial phospholipases accelerate phospholipid release and turnover in the host cell leading to increased COX-2 and PGE_2_ expression [49].

To our knowledge, this study is the first to characterize a post-exposure immunotherapeutic that provides significant protection against lethal *B*. *pseudomallei* pulmonary infection in mice. In an experimental mouse model of tuberculosis, inhibition of PGE_2_ reduced bacillary loads and increased granuloma formation, concomitant with increased IFN-γ, TNF-γ, and iNOS expression, suggesting that PGE_2_ may contribute to *M. tuberculosis* persistence by down-regulation of cell-mediated immunity (CMI) [50]. Similar findings were reported for *F. tularensis* live vaccine strain (LVS) pulmonary challenge in mice [51]. Inhibition of PGE_2_ reduced bacterial loads in the tissues and enhanced CMI responses. COX-2^−/−^ and EP2^−/−^ mice demonstrated accelerated clearance of *Pseudomonas aeruginosa* from the lungs compared to wild type mice, and PGE_2_ signaling via EP2 suppressed macrophage ROS production *in vitro* [41]. COX-2 inhibition also improved bacterial clearance [32], [41] and enhanced host survival [41] during intratracheal infection with *P. aeruginosa*. Collectively, these studies and ours suggest that PGE_2_ production promotes bacterial pathogenesis in the lung and that inhibition of COX-2 may represent a broad-spectrum immunotherapeutic against multiple bacterial pathogens. These results compel further investigation of the role of PGE_2_ in human melioidosis, particularly in patients with pneumonia. Use of commercially-available selective COX-2 inhibitors as an adjunct therapy to antibiotic treatment should also be explored in animal models of melioidosis as combination therapy may further eradicate persistent bacteria.

## Supporting Information

Figure S1 **Lung inflammation is reduced in COX-2 treated mice infected with** ***B. thailandensis*** **.** BALB/c mice were given 15 mg/kg COX-2 inhibitor or mock control and challenged concurrently with 3 LD_50_ *B. thailandensis* by intranasal inoculation. Animals were sacrificed at 48 h post-infection and lungs were stained with H&E. Arrow denotes abundant accumulation of inflammatory cells in mock-treated infected mice. Images obtained at 40X magnification.
(TIFF)

Figure S2 **COX-2 inhibition reduces lung PGE_2_ and tissue bacterial burdens in** ***B*** **.** ***thailandensis-*** **infected mice.** BALB/c mice were given COX-2 inhibitor or mock control and infected i.n. with 3LD_50_ *B. thailandensis*. At 48 h post-infection, mice were sacrificed and lung (A), liver (B), and spleen (C) homogenates plated to determine bacterial cfu. (D) PGE_2_ was measured in lung homogenates by ELISA. * p<0.05 by Mann-Whitney test.
(TIFF)

Table S1 **Fold-change in mRNA expression of 84 different genes from the Toll-like receptor pathway.** J774A.1 macrophages were infected with *B. thailandensis* E264 (MOI 1) and gene expression was analyzed at 2 and 8 hours post-infection. Change in mRNA expression is represented as fold change over uninfected controls. n.c. indicates no change in expression.
(DOCX)

## References

[1] Cheng AC, Currie BJ (2005) Melioidosis: epidemiology, pathophysiology, and management. Clin Microbiol Rev 18: 383–416. 15831829 10.1128/CMR.18.2.383-416.2005 PMC1082802

[2] Larsen JC, Johnson NH (2009) Pathogenesis of *Burkholderia pseudomallei* and *Burkholderia mallei* . Mil Med 174: 647–651. 19585782

[3] Inglis TJJ (2010) The Treatment of Melioidosis. Pharmaceuticals 3: 1296–1303. 27713302 10.3390/ph3051296 PMC4033981

[4] Wiersinga WJ, Currie BJ, Peacock SJ (2012) Melioidosis. N Engl J Med 367: 1035–1044. 22970946 10.1056/NEJMra1204699

[5] Cheng AC, Limmathurotsakul D, Chierakul W, Getchalarat N, Wuthiekanun V, et al (2007) A randomized controlled trial of granulocyte colony-stimulating factor for the treatment of severe sepsis due to melioidosis in Thailand. Clin Infect Dis 45: 308–314. 17599307 10.1086/519261

[6] Goodyear A, Kellihan L, Bielefeldt-Ohmann H, Troyer R, Propst K, et al (2009) Protection from pneumonic infection with burkholderia species by inhalational immunotherapy. Infect Immun 77: 1579–1588. 19179415 10.1128/IAI.01384-08 PMC2663177

[7] Rozak DA, Gelhaus HC, Smith M, Zadeh M, Huzella L, et al (2010) CpG oligodeoxyribonucleotides protect mice from *Burkholderia pseudomallei* but not *Francisella tularensis* Schu S4 aerosols. J Immune Based Ther Vaccines 8: 2. 20181102 10.1186/1476-8518-8-2 PMC2830940

[8] Judy BM, Taylor K, Deeraksa A, Johnston RK, Endsley JJ, et al (2012) Prophylactic application of CpG oligonucleotides augments the early host response and confers protection in acute melioidosis. PLoS One 7: e34176. 22448290 10.1371/journal.pone.0034176 PMC3309019

[9] Easton A, Haque A, Chu K, Patel N, Lukaszewski RA, et al (2011) Combining vaccination and postexposure CpG therapy provides optimal protection against lethal sepsis in a biodefense model of human melioidosis. J Infect Dis 204: 636–644. 21791666 10.1093/infdis/jir301 PMC3144166

[10] Warner TD, Mitchell JA (2004) Cyclooxygenases: new forms, new inhibitors, and lessons from the clinic. FASEB J 18: 790–804. 15117884 10.1096/fj.03-0645rev

[11] Weischenfeldt J, Porse B (2008) Bone Marrow-Derived Macrophages (BMM): Isolation and Applications. CSH Protoc 2008: pdb prot5080. 21356739 10.1101/pdb.prot5080

[12] Burtnick MN, Brett PJ, Nair V, Warawa JM, Woods DE, et al (2008) *Burkholderia pseudomallei* type III secretion system mutants exhibit delayed vacuolar escape phenotypes in RAW 264.7 murine macrophages. Infect Immun 76: 2991–3000. 18443088 10.1128/IAI.00263-08 PMC2446725

[13] Brett PJ, DeShazer D, Woods DE (1998) *Burkholderia thailandensis* sp. nov., a Burkholderia pseudomallei-like species. Int J Syst Bacteriol 48 Pt 1: 317–320. 9542103 10.1099/00207713-48-1-317

[14] Stevens JM, Ulrich RL, Taylor LA, Wood MW, Deshazer D, et al (2005) Actin-binding proteins from *Burkholderia mallei* and *Burkholderia thailandensis* can functionally compensate for the actin-based motility defect of a Burkholderia pseudomallei bimA mutant. J Bacteriol 187: 7857–7862. 16267310 10.1128/JB.187.22.7857-7862.2005 PMC1280302

[15] Haraga A, West TE, Brittnacher MJ, Skerrett SJ, Miller SI (2008) *Burkholderia thailandensis* as a model system for the study of the virulence-associated type III secretion system of Burkholderia pseudomallei. Infect Immun 76: 5402–5411. 18779342 10.1128/IAI.00626-08 PMC2573339

[16] West TE, Frevert CW, Liggitt HD, Skerrett SJ (2008) Inhalation of *Burkholderia thailandensis* results in lethal necrotizing pneumonia in mice: a surrogate model for pneumonic melioidosis. Trans R Soc Trop Med Hyg 102 Suppl 1: S119–126. 19121672 10.1016/S0035-9203(08)70028-2 PMC4764127

[17] Morici LA, Heang J, Tate T, Didier PJ, Roy CJ (2010) Differential susceptibility of inbred mouse strains to *Burkholderia thailandensis* aerosol infection. Microb Pathog 48: 9–17. 19853031 10.1016/j.micpath.2009.10.004 PMC7006035

[18] Ceballos-Olvera I, Sahoo M, Miller MA, Del Barrio L, Re F (2011) Inflammasome-dependent pyroptosis and IL-18 protect against *Burkholderia pseudomallei* lung infection while IL-1beta is deleterious. PLoS Pathog 7: e1002452. 22241982 10.1371/journal.ppat.1002452 PMC3248555

[19] Feterl M, Govan BL, Ketheesan N (2008) The effect of different *Burkholderia pseudomallei* isolates of varying levels of virulence on toll-like-receptor expression. Trans R Soc Trop Med Hyg 102 Suppl 1: S82–88. 19121695 10.1016/S0035-9203(08)70021-X

[20] Wiersinga WJ, Wieland CW, Dessing MC, Chantratita N, Cheng AC, et al (2007) Toll-like receptor 2 impairs host defense in gram-negative sepsis caused by *Burkholderia pseudomallei* (Melioidosis). PLoS Med 4: e248. 17676990 10.1371/journal.pmed.0040248 PMC1950213

[21] West TE, Ernst RK, Jansson-Hutson MJ, Skerrett SJ (2008) Activation of Toll-like receptors by *Burkholderia pseudomallei* . BMC Immunol 9: 46. 18691413 10.1186/1471-2172-9-46 PMC2527550

[22] Wiersinga WJ, Dessing MC, van der Poll T (2008) Gene-expression profiles in murine melioidosis. Microbes Infect 10: 868–877. 18653369 10.1016/j.micinf.2008.04.019

[23] Serezani CH, Chung J, Ballinger MN, Moore BB, Aronoff DM, et al (2007) Prostaglandin E2 suppresses bacterial killing in alveolar macrophages by inhibiting NADPH oxidase. Am J Respir Cell Mol Biol 37: 562–570. 17585108 10.1165/rcmb.2007-0153OC PMC2048683

[24] Miyagi K, Kawakami K, Saito A (1997) Role of reactive nitrogen and oxygen intermediates in gamma interferon-stimulated murine macrophage bactericidal activity against *Burkholderia pseudomallei* . Infect Immun 65: 4108–4113. 9317015 10.1128/iai.65.10.4108-4113.1997 PMC175591

[25] Jones-Carson J, Laughlin JR, Stewart AL, Voskuil MI, Vazquez-Torres A (2012) Nitric oxide-dependent killing of aerobic, anaerobic and persistent *Burkholderia pseudomallei* . Nitric Oxide 10.1016/j.niox.2012.04.001 PMC3517295 22521523

[26] Kalinski P (2012) Regulation of immune responses by prostaglandin E2. J Immunol 188: 21–28. 22187483 10.4049/jimmunol.1101029 PMC3249979

[27] Gotoh T, Mori M (1999) Arginase II downregulates nitric oxide (NO) production and prevents NO-mediated apoptosis in murine macrophage-derived RAW 264.7 cells. J Cell Biol 144: 427–434. 9971738 10.1083/jcb.144.3.427 PMC2132906

[28] Chang CI, Liao JC, Kuo L (2001) Macrophage arginase promotes tumor cell growth and suppresses nitric oxide-mediated tumor cytotoxicity. Cancer Res 61: 1100–1106. 11221839

[29] Das P, Lahiri A, Chakravortty D (2010) Modulation of the arginase pathway in the context of microbial pathogenesis: a metabolic enzyme moonlighting as an immune modulator. PLoS Pathog 6: e1000899. 20585552 10.1371/journal.ppat.1000899 PMC2887468

[30] Warawa JM (2010) Evaluation of surrogate animal models of melioidosis. Front Microbiol 1: 141. 21772830 10.3389/fmicb.2010.00141 PMC3109346

[31] Reddy RC, Chen GH, Tateda K, Tsai WC, Phare SM, et al (2001) Selective inhibition of COX-2 improves early survival in murine endotoxemia but not in bacterial peritonitis. Am J Physiol Lung Cell Mol Physiol 281: L537–543. 11504678 10.1152/ajplung.2001.281.3.L537

[32] Ballinger MN, Aronoff DM, McMillan TR, Cooke KR, Olkiewicz K, et al (2006) Critical role of prostaglandin E2 overproduction in impaired pulmonary host response following bone marrow transplantation. J Immunol 177: 5499–5508. 17015736 10.4049/jimmunol.177.8.5499

[33] Holt DM, Ma X, Kundu N, Collin PD, Fulton AM (2012) Modulation of host natural killer cell functions in breast cancer via prostaglandin E2 receptors EP2 and EP4. J Immunother 35: 179–188. 22306906 10.1097/CJI.0b013e318247a5e9 PMC3721982

[34] Nokta MA, Pollard RB (1992) Human immunodeficiency virus replication: modulation by cellular levels of cAMP. AIDS Res Hum Retroviruses 8: 1255–1261. 1381600 10.1089/aid.1992.8.1255

[35] Vancheri C, Mastruzzo C, Sortino MA, Crimi N (2004) The lung as a privileged site for the beneficial actions of PGE2. Trends Immunol 25: 40–46. 14698283 10.1016/j.it.2003.11.001

[36] Peskar BM, Sawka N, Ehrlich K, Peskar BA (2003) Role of cyclooxygenase-1 and -2, phospholipase C, and protein kinase C in prostaglandin-mediated gastroprotection. J Pharmacol Exp Ther 305: 1233–1238. 12626649 10.1124/jpet.103.049650

[37] Ozaki T, Rennard SI, Crystal RG (1987) Cyclooxygenase metabolites are compartmentalized in the human lower respiratory tract. J Appl Physiol 62: 219–222. 3104286 10.1152/jappl.1987.62.1.219

[38] Wiersinga WJ, van der Poll T (2009) Immunity to B*urkholderia pseudomallei* . Curr Opin Infect Dis 22: 102–108. 19276877 10.1097/QCO.0b013e328322e727

[39] Skyberg JA, Rollins MF, Holderness JS, Marlenee NL, Schepetkin IA, et al (2012) Nasal Acai polysaccharides potentiate innate immunity to protect against pulmonary *Francisella tularensis* and *Burkholderia pseudomallei* Infections. PLoS Pathog 8: e1002587. 22438809 10.1371/journal.ppat.1002587 PMC3305411

[40] Harbrecht BG, Kim YM, Wirant EA, Simmons RL, Billiar TR (1997) Timing of prostaglandin exposure is critical for the inhibition of LPS- or IFN-gamma-induced macrophage NO synthesis by PGE2. J Leukoc Biol 61: 712–720. 9201262 10.1002/jlb.61.6.712

[41] Sadikot RT, Zeng H, Azim AC, Joo M, Dey SK, et al (2007) Bacterial clearance of *Pseudomonas aeruginosa* is enhanced by the inhibition of COX-2. Eur J Immunol 37: 1001–1009. 17330822 10.1002/eji.200636636

[42] Marotta P, Sautebin L, Di Rosa M (1992) Modulation of the induction of nitric oxide synthase by eicosanoids in the murine macrophage cell line J774. Br J Pharmacol 107: 640–641. 1282071 10.1111/j.1476-5381.1992.tb14499.x PMC1907741

[43] Kunkel SL, Wiggins RC, Chensue SW, Larrick J (1986) Regulation of macrophage tumor necrosis factor production by prostaglandin E2. Biochem Biophys Res Commun 137: 404–410. 3459461 10.1016/0006-291x(86)91224-6

[44] Rodriguez PC, Hernandez CP, Quiceno D, Dubinett SM, Zabaleta J, et al (2005) Arginase I in myeloid suppressor cells is induced by COX-2 in lung carcinoma. J Exp Med 202: 931–939. 16186186 10.1084/jem.20050715 PMC2213169

[45] Haas AR, Sun J, Vachani A, Wallace AF, Silverberg M, et al (2006) Cycloxygenase-2 inhibition augments the efficacy of a cancer vaccine. Clin Cancer Res 12: 214–222. 16397045 10.1158/1078-0432.CCR-05-1178

[46] Zhang Z, Reenstra W, Weiner DJ, Louboutin JP, Wilson JM (2007) The p38 mitogen-activated protein kinase signaling pathway is coupled to Toll-like receptor 5 to mediate gene regulation in response to *Pseudomonas aeruginosa* infection in human airway epithelial cells. Infect Immun 75: 5985–5992. 17908812 10.1128/IAI.00678-07 PMC2168327

[47] Uematsu S, Jang MH, Chevrier N, Guo Z, Kumagai Y, et al (2006) Detection of pathogenic intestinal bacteria by Toll-like receptor 5 on intestinal CD11c+ lamina propria cells. Nat Immunol 7: 868–874. 16829963 10.1038/ni1362

[48] Hawn TR, Berrington WR, Smith IA, Uematsu S, Akira S, et al (2007) Altered inflammatory responses in TLR5-deficient mice infected with *Legionella pneumophila* . J Immunol 179: 6981–6987. 17982089 10.4049/jimmunol.179.10.6981

[49] Titball RW (1993) Bacterial phospholipases C. Microbiol Rev 57: 347–366. 8336671 10.1128/mr.57.2.347-366.1993 PMC372913

[50] Rangel Moreno J, Estrada Garcia I, De La Luz Garcia Hernandez M, Aguilar Leon D, Marquez R, et al (2002) The role of prostaglandin E2 in the immunopathogenesis of experimental pulmonary tuberculosis. Immunology 106: 257–266. 12047755 10.1046/j.1365-2567.2002.01403.x PMC1782721

[51] Woolard MD, Hensley LL, Kawula TH, Frelinger JA (2008) Respiratory *Francisella tularensis* live vaccine strain infection induces Th17 cells and prostaglandin E2, which inhibits generation of gamma interferon-positive T cells. Infect Immun 76: 2651–2659. 18391003 10.1128/IAI.01412-07 PMC2423094

