# Supplementary figures and images for: Post-Exposure Therapeutic Efficacy of COX-2 Inhibition against Burkholderia pseudomallei
Source: PLoS Negl Trop Dis. 2013 May 9;7(5):e2212. doi: 10.1371/journal.pntd.0002212 (PMC3649956; doi:10.1371/journal.pntd.0002212)

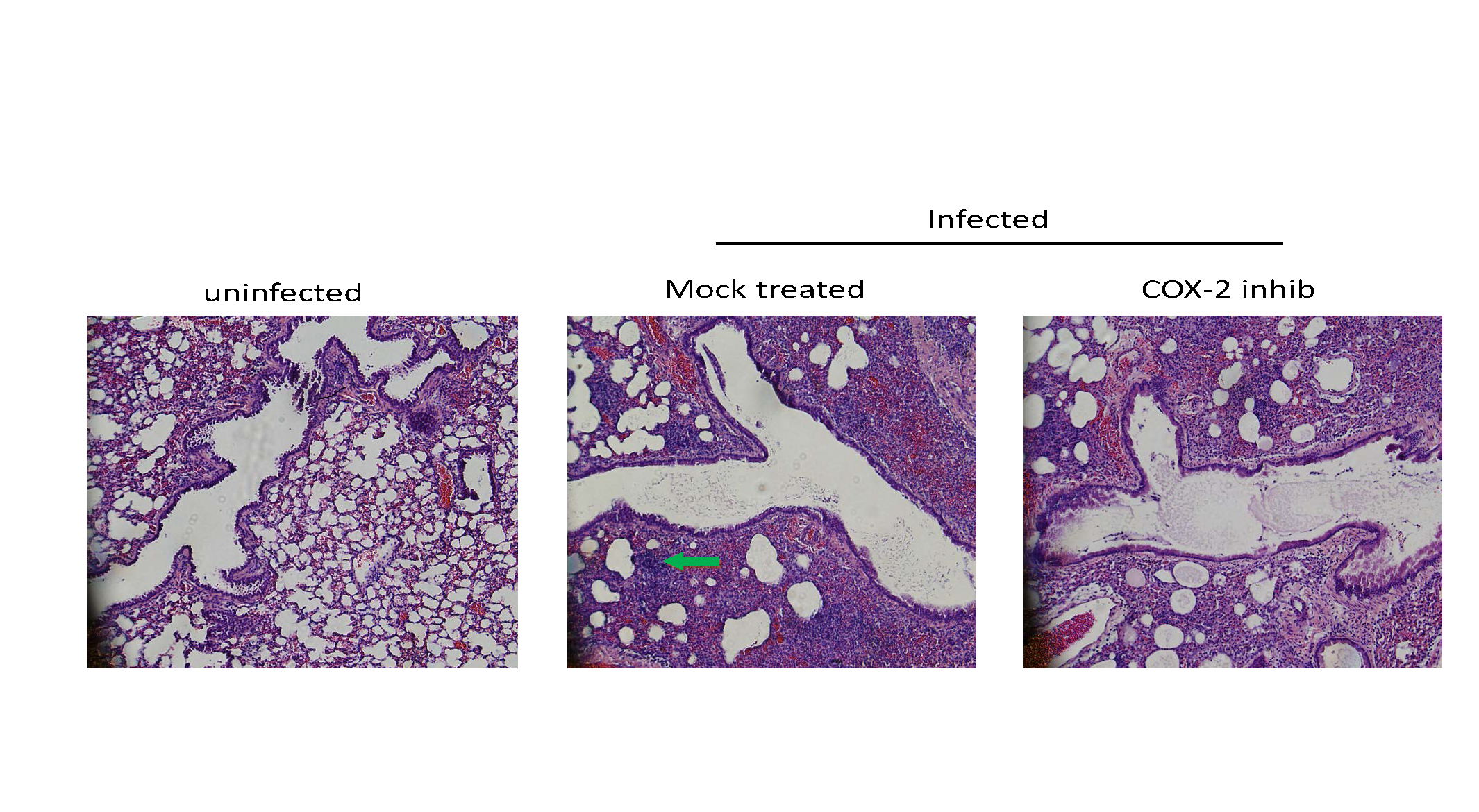

Supplement: Figure S1 — Lung inflammation is reduced in COX-2 treated mice infected with B. thailandensis . BALB/c mice were given 15 mg/kg COX-2 inhibitor or mock control and challenged concurrently with 3 LD50 B. thailandensis by intranasal inoculation. Animals were sacrificed at 48 h post-infection and lungs were stained with H&E. Arrow denotes abundant accumulation of inflammatory cells in mock-treated infected mice. Images obtained at 40X magnification. (TIFF) [file pntd.0002212.s001.tiff]
