# Supplementary material for: Post-Exposure Therapeutic Efficacy of COX-2 Inhibition against Burkholderia pseudomallei
Source: PLoS Negl Trop Dis. 2013 May 9;7(5):e2212. doi: 10.1371/journal.pntd.0002212 (PMC3649956; doi:10.1371/journal.pntd.0002212)
Supplement: Table S1 — Fold-change in mRNA expression of 84 different genes from the Toll-like receptor pathway. J774A.1 macrophages were infected with B. thailandensis E264 (MOI 1) and gene expression was analyzed at 2 and 8 hours post-infection. Change in mRNA expression is represented as fold change over uninfected controls. n.c. indicates no change in expression. (DOCX) [file pntd.0002212.s003.docx]

| **Symbol** | **Description** | **Fold-Difference 2h** | **Fold-Difference 8h** |
| --- | --- | --- | --- |
| Agfg1 | ArfGAP with FG repeats 1 | n.c. | 128.0 |
| Btk | Bruton agammaglobulinemia tyrosine kinase | n.c. | n.c. |
| Casp8 | Caspase 8 | n.c. | n.c. |
| Ccl2 | Chemokine (C-C motif) ligand 2 | 5.0 | 13.9 |
| Cd14 | CD14 antigen | n.c. | 4.5 |
| Cd80 | CD80 antigen | n.c. | 17.1 |
| Cd86 | CD86 antigen | n.c. | 3821.7 |
| Cebpb | CCAAT/enhancer binding protein (C/EBP), beta | n.c. | 24.2 |
| Chuk | Conserved helix-loop-helix ubiquitous kinase | -5.4 | 1448.1 |
| Clec4e | C-type lectin domain family 4, member e | n.c. | 274.3 |
| Csf2 | Colony stimulating factor 2 (granulocyte-macrophage) | 4.4 | 24.2 |
| Csf3 | Colony stimulating factor 3 (granulocyte) | 66.2 | 5042.7 |
| Cxcl10 | Chemokine (C-X-C motif) ligand 10 | 30.9 | 8.0 |
| Eif2ak2 | Eukaryotic translation initiation factor 2-alpha kinase 2 | 7.2 | 445.7 |
| Elk1 | ELK1, member of ETS oncogene family | n.c. | 5.2 |
| Fadd | Fas (TNFRSF6)-associated via death domain | n.c. | n.c. |
| Fos | FBJ osteosarcoma oncogene | n.c. | 34.2 |
| Hmgb1 | High mobility group box 1 | -5.8 | n.c. |
| Hras1 | Harvey rat sarcoma virus oncogene 1 | n.c. | n.c. |
| Hspa1a | Heat shock protein 1A | n.c. | n.c. |
| Hspd1 | Heat shock protein 1 (chaperonin) | n.c. | 7643.4 |
| Ifnb1 | Interferon beta 1, fibroblast | 5.8 | 8.0 |
| Ifng | Interferon gamma | n.c. | 8.0 |
| Ikbkb | Inhibitor of kappaB kinase beta | n.c. | 55.7 |
| Il10 | Interleukin 10 | 10.1 | 97.0 |
| Il12a | Interleukin 12A | n.c. | 8.0 |
| Il1a | Interleukin 1 alpha | 21.8 | 103.9 |
| Il1b | Interleukin 1 beta | 26.9 | 194.0 |
| Il1r1 | Interleukin 1 receptor, type I | n.c. | n.c. |
| Il2 | Interleukin 2 | n.c. | n.c. |
| Il6 | Interleukin 6 | 76.1 | 891.4 |
| Il6ra | Interleukin 6 receptor, alpha | n.c. | n.c. |
| Irak1 | Interleukin-1 receptor-associated kinase 1 | -5.0 | 73.5 |
| Irak2 | Interleukin-1 receptor-associated kinase 2 | n.c. | 2702.3 |
| Irf1 | Interferon regulatory factor 1 | n.c. | 207.9 |
| Irf3 | Interferon regulatory factor 3 | n.c. | 25.9 |
| Jun | Jun oncogene | n.c. | 1552.0 |
| Lta | Lymphotoxin A | n.c. | n.c. |
| Ly86 | Lymphocyte antigen 86 | -13.4 | n.c. |
| Ly96 | Lymphocyte antigen 96 | n.c. | n.c. |
| Map2k3 | Mitogen-activated protein kinase kinase 3 | n.c. | n.c. |
| Map2k4 | Mitogen-activated protein kinase kinase 4 | n.c. | 36.7 |
| Map3k1 | Mitogen-activated protein kinase kinase kinase 1 | n.c. | 8.0 |
| Map3k7 | Mitogen-activated protein kinase kinase kinase 7 | n.c. | 831.7 |
| Mapk8 | Mitogen-activated protein kinase 8 | n.c. | 128 |
| Mapk8ip3 | Mitogen-activated protein kinase 8 interacting protein 3 | n.c. | 1024 |
| Mapk9 | Mitogen-activated protein kinase 9 | n.c. | 55.7 |
| Muc13 | Mucin 13, epithelial transmembrane | -8.2 | 8 |
| Myd88 | Myeloid differentiation primary response gene 88 | n.c. | 18.3 |
| Nfkb1 | Nuclear factor of kappa light polypeptide gene enhancer in B-cells 1, p105 | 8.8 | n.c. |
| Nfkb2 | Nuclear factor of kappa light polypeptide gene enhancer in B-cells 2, p49/p100 | n.c. | 8 |
| Nfkbia | Nuclear factor of kappa light polypeptide gene enhancer in B-cells inhibitor, alpha | 6.2 | 19.6 |
| Nfkbib | Nuclear factor of kappa light polypeptide gene enhancer in B-cells inhibitor, beta | n.c. | 8 |
| Nfkbil1 | Nuclear factor of kappa light polypeptide gene enhancer in B-cells inhibitor-like 1 | n.c. | n.c. |
| Nfrkb | Nuclear factor related to kappa B binding protein | n.c. | 18.3 |
| Nr2c2 | Nuclear receptor subfamily 2, group C, member 2 | n.c. | 5.6 |
| Peli1 | Pellino 1 | n.c. | 3326.9 |
| Pglyrp1 | Peptidoglycan recognition protein 1 | n.c. | 8 |
| Ppara | Peroxisome proliferator activated receptor alpha | n.c. | 22.6 |
| Ptgs2 (COX-2) | Prostaglandin-endoperoxide synthase 2 | 430.5 | 16384 |
| Rel | Reticuloendotheliosis oncogene | 8.2 | n.c. |
| Rela | V-rel reticuloendotheliosis viral oncogene homolog A (avian) | n.c. | n.c. |
| Ripk2 | Receptor (TNFRSF)-interacting serine-threonine kinase 2 | n.c. | 6.4 |
| Tbk1 | TANK-binding kinase 1 | n.c. | n.c. |
| Ticam1 | Toll-like receptor adaptor molecule 1 | n.c. | n.c. |
| Ticam2 | Toll-like receptor adaptor molecule 2 | n.c. | 16 |
| Tirap | Toll-interleukin 1 receptor (TIR) domain-containing adaptor protein | 6.7 | 5.6 |
| Tlr1 | Toll-like receptor 1 | n.c. | 84.4 |
| Tlr2 | Toll-like receptor 2 | n.c. | 2194.9 |
| Tlr3 | Toll-like receptor 3 | n.c. | 42.2 |
| Tlr4 | Toll-like receptor 4 | 5.8 | 207.9 |
| Tlr5 | Toll-like receptor 5 | n.c. | 7.4 |
| Tlr6 | Toll-like receptor 6 | n.c. | n.c. |
| Tlr7 | Toll-like receptor 7 | n.c. | -4.5 |
| Tlr8 | Toll-like receptor 8 | n.c. | -8 |
| Tlr9 | Toll-like receptor 9 | n.c. | -6.4 |
| Tnf | Tumor necrosis factor | 7.2 | 7.4 |
| Tnfaip3 | Tumor necrosis factor, alpha-induced protein 3 | 5.8 | 42.2 |
| Tnfrsf1a | Tumor necrosis factor receptor superfamily, member 1a | 76.1 | n.c. |
| Tollip | Toll interacting protein | n.c. | 9.1 |
| Tradd | TNFRSF1A-associated via death domain | n.c. | 181.0 |
| Traf6 | Tnf receptor-associated factor 6 | 28.8 | 588.1 |
| Ube2n | Ubiquitin-conjugating enzyme E2N | 10.9 | 32 |
| Ube2v1 | Ubiquitin-conjugating enzyme E2 variant 1 | n.c. | 256 |
